# Effects of carbohydrates-BCAAs-caffeine ingestion on performance and neuromuscular function during a 2-h treadmill run: a randomized, double-blind, cross-over placebo-controlled study

**DOI:** 10.1186/1550-2783-8-22

**Published:** 2011-12-07

**Authors:** Sébastien L Peltier, Lucile Vincent, Guillaume Y Millet, Pascal Sirvent, Jean-Benoît Morin, Michel Guerraz, André Geyssant, Jean-François Lescuyer, Léonard Feasson, Laurent Messonnier

**Affiliations:** 1 Laboratoire Lescuyer, Aytré, France; 2 Exercise Physiology Laboratory, Department of Sport Sciences, University of Savoie, F-73376 Le Bourget du Lac Cedex France; 3 Université de Lyon, F-42023, Saint-Etienne, France; 4 Clermont Université, Université Blaise Pascal, EA 3533, Laboratoire des Adaptations Métaboliques à l'Exercice en conditions Physiologiques et Pathologiques (AME2P), BP 80026, F-63171 Aubière Cedex, France; 5 Laboratory of Psychology and Neurocognition (UMR 5105), University of Savoie, 73000 Chambéry, France

## Abstract

**Background:**

Carbohydrates (CHOs), branched-chain amino acids (BCAAs) and caffeine are known to improve running performance. However, no information is available on the effects of a combination of these ingredients on performance and neuromuscular function during running.

**Methods:**

The present study was designed as a randomized double-blind cross-over placebo-controlled trial. Thirteen trained adult males completed two protocols, each including two conditions: placebo (PLA) and Sports Drink (SPD: CHOs 68.6 g.L^-1^, BCAAs 4 g.L^-1^, caffeine 75 mg.L^-1^). Protocol 1 consisted of an all-out 2 h treadmill run. Total distance run and glycemia were measured. In protocol 2, subjects exercised for 2 h at 95% of their lowest average speeds recorded during protocol 1 (whatever the condition). Glycemia, blood lactate concentration and neuromuscular function were determined immediately before and after exercise. Oxygen consumption ($\dot{\text{V}}{\text{O}}_{2}$), heart rate (HR) and rate of perceived exertion (RPE) were recorded during the exercise. Total fluids ingested were 2 L whatever the protocols and conditions.

**Results:**

Compared to PLA, ingestion of SPD increased running performance (p = 0.01), maintained glycemia and attenuated central fatigue (p = 0.04), an index of peripheral fatigue (p = 0.04) and RPE (p = 0.006). Maximal voluntary contraction, $\dot{\text{V}}{\text{O}}_{2}$, and HR did not differ between the two conditions.

**Conclusions:**

This study showed that ingestion of a combination of CHOs, BCAAs and caffeine increased performance by about 2% during a 2-h treadmill run. The results of neuromuscular function were contrasted: no clear cut effects of SPD were observed.

**Trial registration:**

ClinicalTrials.gov, http://www.clinicaltrials.gov, NCT00799630

## Background

Prolonged running exercises may induce hypoglycemia, central and/or peripheral fatigue, muscle damage, osteoarticular disorders, inflammation and cardiovascular dysfunction [1-4]. An adapted carbohydrate (CHO) supplement during exercise may be useful for limiting and/or avoiding hypoglycemia and the associated disturbance of physical ability. Previous experiments have shown that ingested CHOs improve performance during exercise of longer than ~45 min [5-7]. However, the observed improvement varies and depends, among other things, on CHO dosage, exercise intensity and duration, and the training status of the subjects [8,9]. For example, Coyle showed that during a prolonged strenuous cycling exercise (71 ± 1% V˙O2 max) fatigue occurred after 3.02 ± 0.19 h in a placebo trial *versus *4.02 ± 0.33 h in a CHO supplement trial (glucose polymer solution, 2.0 g.kg^-1 ^at 20 min and 0.4 g.kg^-1 ^every 20 min thereafter) [5]. During a cycling time trial, Jeukendrup et al. [6] observed that the time needed to complete the set amount of work was significantly shorter with CHOs (7.6%) than with the placebo (58.7 ± 0.5 min *versus *60.2 ± 0.7 min, respectively), corresponding to a higher percentage of the subjects' maximal work rate. It should be noted that increased performance is not systematically observed with CHO ingestion [10]. The mechanisms for the beneficial effect of CHOs on performance are thought to be via the maintenance of plasma glucose concentrations and the high rates of exogenous CHO oxidation in the latter stages of exercise when muscle and liver glycogen levels are low [5,11,12].

A great deal of research has been conducted to test different combinations of CHOs and their exogenous oxidation. In particular, studies have demonstrated that blends of simple carbohydrates containing fructose and sucrose, glucose, maltose, galactose or maltodextrins promote greater exogenous glucose oxidation than do isocaloric glucose solutions. The difference is thought to be due, at least in part, to the recruitment of multiple intestinal sugar transporters (sodium glucose transporter-1 and GLUT-5) [13-16]. During exercise, the ingested glucose is rapidly absorbed into the circulation and oxidized by the skeletal muscle in a highly efficient manner. In contrast, ingestion of fructose and galactose results in less efficient oxidization probably related to slower absorption and delays linked to hepatic metabolism [17-19]. Nevertheless, when ingested at a rate designed to saturate intestinal CHO transport systems, fructose and galactose enhance postexercise human liver glycogen synthesis [20].

Caffeine can also be used to extend endurance exercise and improve performance. Kovacs et al. [21] identified improvements in performance during cycling time trials when moderate amounts of caffeine (2.1 and 4.5 mg.kg^-1^) were ingested in combination with a 7% CHO solution during exercise. This effect may be partly explained by the fact that a caffeine-glucose combination increases exogenous CHO oxidation more than does glucose alone, possibly as a result of enhanced intestinal absorption [22]. It is also possible that the caffeine causes a decrease in central fatigue [23]. In fact caffeine can block adenosine receptors even at concentrations in the micromolar range [23]. Stimulation of adenosine receptors induces an inhibitory effect on central excitability.

Another interesting nutritional strategy to improve performance is the ingestion of branched-chain amino acids (BCAAs, *i.e*., leucine, isoleucine and valine) during exercise. Blomstrand et al. [24] suggested that an intake of BCAAs (7.5 - 12 g) during exercise can prevent or decrease the net rate of protein degradation caused by heavy exercise. Moreover, BCAAs supply during exercise might have a sparing effect on muscle glycogen degradation [25]. It has also been postulated that BCAAs supply during prolonged exercise might reduce central fatigue [4]. Fatigue is generally defined as the inability to maintain power output [26], and can be central and/or peripheral in its origin, these two factors being interrelated. Several factors have been identified as a cause of peripheral fatigue (*e.g*., the action potential transmission along the sarcolemma, excitation-contraction coupling (E-C), actin-myosin interaction), whereas the factors underlying central fatigue could be located at the spinal and/or supraspinal sites. The tryptophan-5-hydroxytryptamine-central fatigue theory has been proposed to explain how oral administration of BCAAs can attenuate central fatigue [26]. During prolonged aerobic exercise, the concentration of free tryptophan, and thus the uptake of tryptophan into the brain, increases. When this occurs, 5-hydroxytryptamine (5-HT, serotonin) is produced, which has been postulated to play a role in the subjective feelings of fatigue. Because BCAAs are transported into the brain by the same carrier system as tryptophan, increasing BCAAs plasma concentration may decrease the uptake of tryptophan in the brain, and consequently the feeling of fatigue. Nevertheless, Meeusen et al. [27] have mentioned that brain function is not determined by a single neurotransmitter system and the interaction between brain serotonin and dopamine during prolonged exercise has also been explored as having a regulatory role in the development of fatigue. Hence, Meeusen et al. [27] suggest that an increase in the central ratio of serotonin to dopamine is associated with feelings of tiredness and lethargy. Consequently, it cannot be excluded that the given role of serotonin in the development of central fatigue is overestimated. Nevertheless, taken together these data suggest that BCAAs supplements taken during prolonged exercise may have beneficial effects on some of the metabolic causes of fatigue such as glycogen depletion and central fatigue.

Consequently it is likely that a beverage containing a mixture of CHOs, caffeine and BCAAs would improve an athlete's performance during endurance exercise. To our knowledge, no information is available on the effects of this combination on physical performance and neuromuscular function. The main purpose of the present study was therefore to investigate whether ingestion of an association of CHOs (68.6 g.L^-1^), BCAAs (4 g.L^-1^) and caffeine (75 mg.L^-1^) is efficient in improving physical performance and limiting alterations to neuromuscular function during a prolonged running exercise.

## Methods

### Subjects

Subject data are documented in Table 1. The subjects regularly trained at least 2 - 4 times per week and had been involved in endurance training and competition for at least 3 months. All subjects were habitual caffeine users (1 - 2 cups of coffee or equivalent per day). Before participation, each subject was fully informed of the purpose and risks associated with the procedures, and their written informed consent was obtained. All subjects were healthy, as assessed by a medical examination. The study was approved by the Southeast Ethics Committee for Human Research (France, ClinicalTrials.gov, http://www.clinicaltrials.gov, NCT00799630).

**Table 1:** Main characteristics of the subjects

Age (yr)	Body mass (kg)	Height (cm)	BMI (kg.m^-2^)	Body Fat (%)	V˙O2 max**(mL.min^-1^.kg^-1^)**
29.6 ± 9.2	71.7 ± 5.1	179.2 ± 5.7	22.4 ± 2.1	14.0 ± 3.3	59.7 ± 4.8

V˙O2 max, maximal oxygen uptake; BMI: Body mass index.

Values are means ± SD.

### Preliminary testing

At least 1 week before the start of the experimental trials, an incremental exercise test to volitional exhaustion was performed on a treadmill. This graded exercise aimed i) to check the tolerance of the subjects to maximal exercise, ii) to characterize their physical fitness, and iii) to familiarize the subjects to the use of the treadmill and the experimental procedures. After a gentle warm-up, the test started at 10 km.h^-1^, and velocity was then increased by 1.5 km.h^-1 ^every 3 min. Oxygen uptake ($\dot{\text{V}}{\text{O}}_{2}$) was measured during the last minute of each 3-min period of the maximal incremental test as presented elsewhere [28]. Briefly, subjects breathed through a two-way non-rebreathing valve (series 2700, Hans Rudolph, Kansas City, Missouri, USA) connected to a three-way stopcock for the collection of gases (100 L bag). The volume of the expired gas was measured in a Tissot spirometer (Gymrol, Roche-la-Molière, France). Fractions of expired gases were determined with a paramagnetic O_2 _analyzer (Servomex, cell 1155B, Crowborough, England) and infrared CO_2 _analyzer (Normocap Datex). The analyzers were calibrated with mixed gases, the composition of which was determined using Scholander's method [29]. Heart rate (HR) was recorded continuously by a radio telemetry HR monitor (S810, Polar^®^, Tampere, Finland). Individual maximal oxygen uptake (V˙O2 max) was determined as previously described [30].

### Experimental design

The study was designed as a randomized double-blind cross-over placebo-controlled trial. The random allocation sequences were generated by an automated system under the supervision of the committee of protection of human subjects. The codes were kept confidential until the end of the study when the randomisation code was broken. All the subjects and investigators were blind to the randomisation codes throughout the study.

**Figure 1:**
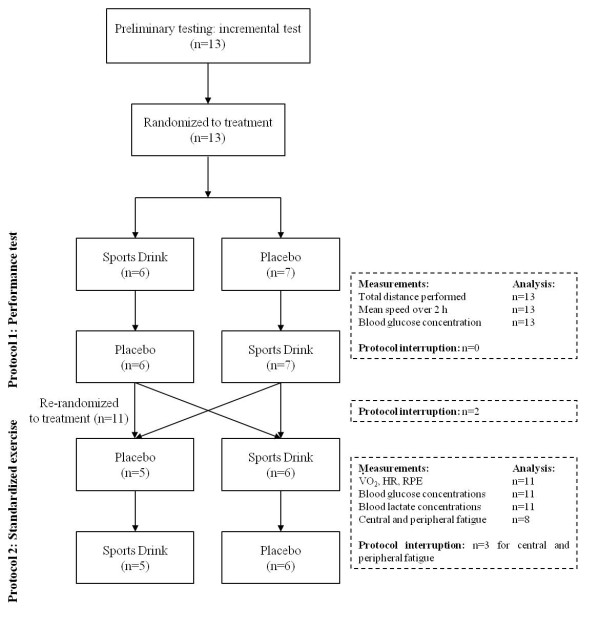
**Experimental design and diagram of flow of subjects through the study protocol**. $\dot{\text{V}}{\text{O}}_{2}$: oxygen consumption; RER: Respiratory Exchange Ratio; HR: heart rate; RPE: rate of perceived exertion.

The experiment comprised two exercise protocols, each of them including two exercise tests performed in different conditions: *i.e*., with ingestion of the sports drink (SPD) or with a placebo (PLA) (see Protocols and Figure 1 for details). The two exercise tests in protocol 1 were completed in randomized order at least one week apart. At least one week following protocol 1, protocol 2 began. As for protocol 1, the exercise tests in protocol 2 were performed in randomized order at least one week apart. Subjects were instructed to maintain their usual daily exercise activity and dietary intake (in particular, their caffeine intake) during the study but not to consume any solid or liquid nutrients with the exception of water for 2 h before each exercise session. All the exercises performed by any one subject were done at the same time of the day. The subjects were instructed to replicate the same meal before each exercise session.

### Protocol 1: Performance test

Before the exercise, a 20 μL blood sample was collected from an earlobe for the assessment of resting blood glucose concentration. Then, in the 15 min preceding the test, the subjects drank 250 mL of one of the two drinks (PLA or SPD). Thereafter, the running test started by a gentle warm-up followed by a 2 hour all-out exercise trial. A beverage volume of 250 mL was provided every 15 min and drunk by the subjects within the next 15 min so that the total fluids ingested before and during the 2-hour exercise was 2 liters. The volume and kinetics of beverage ingestion was chosen to minimize dehydration [16] and gastrointestinal discomfort. The subjects ran without knowing their actual speed. An experimenter changed the velocity of the treadmill following each subject's recommendations so that they could give their best performance during the 2-hour exercise. At the end of the exercise a second blood sample was collected for glucose determination. Total distance (km) was recorded and average speed (km.h^-1^) was calculated. Total distance (unknown by the subject) was considered as physical performance.

### Protocol 2: Standardized exercise

A 20 μL blood sample was collected from the earlobe for the assessment of resting glucose and lactate concentrations. As in protocol 1, 15 min before the test and just before their gentle warm-up subjects drank 250 mL of PLA or SPD. Thereafter, the subjects exercised for 2 hours at 95% of their individual lowest average speed sustained in PLA or SPD during protocol 1; 250 mL of beverage was provided every 15 min. During exercise, $\dot{\text{V}}{\text{O}}_{2}$, $\dot{\text{V}}{\text{CO}}_{2}$, Respiratory Exchange Ratio (RER: $\dot{\text{V}}{\text{CO}}_{2}$/$\dot{\text{V}}{\text{O}}_{2}$), HR and Rate of Perceived Exertion (RPE) were measured and/or recorded every 20 min. Central and peripheral fatigue was evaluated before and immediately after exercise.

### Material and procedures

All exercises were performed on the same treadmill (EF 1800, HEF Tecmachine, Andrezieux-Boutheon, France). Blood lactate and glucose concentrations were determined enzymatically using a YSI 2300 (Yellow Spring Instrument, USA). $\dot{\text{V}}{\text{O}}_{2}$ and $\dot{\text{V}}{\text{CO}}_{2}$ were measured as described above (see paragraph *Preliminary testing*). RPE was determined using the 6 - 20 point Borg scale [31].

### Central and peripheral fatigue measurements

Tests were performed on the knee extensors. The subjects were seated in the frame of a Cybex II (Ronkonkoma, NY) and Velcro straps were used to limit lateral and frontal displacements. The subjects were instructed to grip the seat during the voluntary contractions to stabilize the pelvis. The knee extensor muscles' mechanical response was recorded with a strain gauge (SBB 200 Kg, Tempo Technologies, Taipei, Taiwan). All measurements were taken from the subject's right leg, with the knee and hip flexed at 90 degrees from full extension. The isometric contractions performed during the experiment included 3-4-s maximal voluntary contractions and electrically evoked contractions. During the 4 MVCs, the subjects were strongly encouraged. Femoral nerve electrical stimulation was performed using a cathode electrode (10-mm diameter, Ag-AgCl, Type 0601000402, Contrôle Graphique Medical, Brie-Comte-Robert, France) pressed over the femoral nerve in the femoral triangle, 3-5 cm below the inguinal ligament with the anode (10.2 cm × 5.2 cm, Compex, SA, Ecublens, Switzerland) placed over the gluteal fold. Electrical impulses (single, square-wave, 1-ms duration) were delivered with a constant current, high-voltage (maximal voltage 400 V) stimulator (Digitimer, DS7A, Hertfordshire, UK). For all stimulus modalities, stimulation intensity corresponded to ~120% of optimal intensity, *i.e*. the stimulus intensity at which the maximal amplitude of both twitch force and the concomitant *vastus lateralis *(VL) M wave (see below) were reached.

The surface electromyographic (EMG) signal was recorded from the right VL muscle with two pairs of bipolar oval self-adhesive electrodes with an inter electrode distance of 2.5 cm (10 mm diameter, Ag-AgCl, Type 0601000402, Contrôle Graphique Medical, Brie-Comte-Robert, France). The position and placement of the electrodes followed SENIAM recommendations. EMG data were recorded with the PowerLab system 16/30 - ML880/P (ADInstruments, Sydney, Australia) at a sample frequency of 2000 Hz. The EMG signals were amplified with an octal bio amplifier - ML138 (ADInstruments) with bandwidth frequency ranging from 3 Hz to 1 kH (input impedance = 200 MΩ, common mode rejection ratio = 85 dB, gain = 1000), transmitted to a PC and analyzed with LabChart6 software (ADInstruments).

The twitch interpolation technique was used to determine potential change in maximal voluntary activation [32]. This consisted in superimposing stimulation at supramaximal intensity on the isometric plateau of a maximal voluntary contraction of the knee extensors. In this study a high-frequency paired stimulation (doublet at 100 Hz, Db_100_) was used instead of a single twitch. A second 100 Hz doublet (control stimulation) was delivered to the relaxed muscle 3 s after the end of the contraction. This provided the opportunity to obtain a potentiated mechanical response and so reduce variability in activation level (%VA) values. The ratio of the amplitude of the superimposed doublet over the size of the control doublet was then calculated to obtain voluntary activation (%VA) as follows:

$$\%\text{VA}=\left(\text{1}-\left(\text{Superimposed}\;\text{D}{\text{b}}_{\text{1}00}\text{torque}∕\text{Mean}\;\text{control}\;\text{D}{\text{b}}_{\text{1}00}\text{torque}\right)\right)\times \text{1}00$$

Three MVCs separated by 30 s, were performed to determine MVC and %VA. The quadriceps muscle's isometric twitch peak torque and contraction time and VL M-wave peak-to-peak amplitude and duration were also analyzed. To do this, three potentiated single twitches were evoked after a 4^th ^MVC and averaged. %VA changes were considered as indices of central fatigue. Changes in electrically evoked contraction of the relaxed muscle (high-frequency doublet mechanical response, peak twitch) were the outcome measures for peripheral fatigue.

### Composition of drinks

The doses of CHOs, BCAAs and caffeine were chosen to be as close as possible to those used in previous studies [12,15,21,33,34] and the palatability of the sports drink. For instance, due to the bitter taste of BCAAs, it is difficult to incorporate more than 4 g.L^-1 ^of these amino acids in a drink. Moreover, theses doses respect the current legislation for dietary products. The nutritional composition of SPD was as follows: maltodextrin 31.6 g.L^-1^, dextrose 24.2 g.L^-1^, fructose 12.8 g.L^-1^, branched-chain amino acids 4 g.L^-1^, curcumin 250 mg.L^-1^, piperine 2.6 mg.L^-1^, caffeine 75 mg.L^-1^, sodium 884 mg.L^-1^, magnesium 100 mg.L^-1^, zinc 5 mg.L^-1^, vitamins C 15 mg.L^-1^, E 5 mg.L^-1^, B1 0.7 mg.L^-1^, B2 0.4 mg.L^-1^, B3 9 mg.L^-1^. Composition of the PLA drink: malic and citric acids, xanthan gum, acesulfame potassium, sucralose, silicium dioxide, yellow FCF, tartrazine. The energy provided by SPD and PLA was 1254 and 50 kJ.L^-1 ^respectively. SPD and PLA were provided by Nutratletic (Aytre, France).

### Statistical analysis

The results are presented as mean values ± SD. Because of the lack of normality, data describing running performance, blood glucose and lactate concentrations and neuromuscular variables obtained in the two conditions were compared using the non-parametric Wilcoxon test. $\dot{\text{V}}{\text{O}}_{2}$, RER, HR, and RPE were subjected to a two-way repeated-measure analysis of variance describing the effect of drink ingestion (PLA and SPD) (external factor), exercise duration (internal factor) and their interaction. A p-value < 0.05 was considered as significant.

## Results

### Protocol 1: Performance test

**Figure 2:**
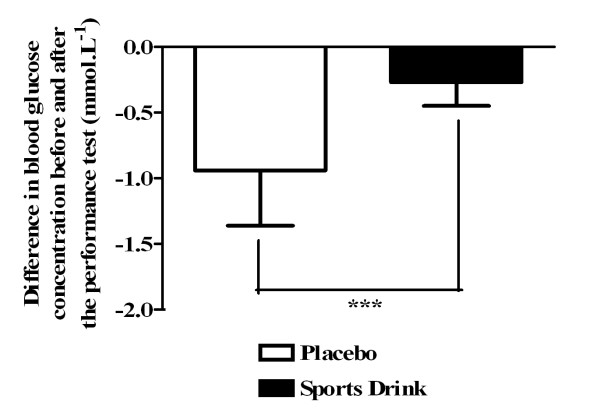
**Difference in blood glucose concentration before and after the performance test (protocol 1)**. Values are means ± SD. *** p = 0.0002.

Running distance was significantly higher, *i.e*. performance was better, in SPD than in PLA (22.31 ± 1.85 *vs*. 21.90 ± 1.69 km, n = 13, p = 0.01). Before exercise, there was no difference in mean glucose concentrations between PLA and SPD (5.60 ± 0.82 and 5.53 ± 0.85 mmol.L^-1^, respectively, n = 13, NS). After exercise, blood glucose was significantly lower than before exercise in both groups (4.66 ± 0.48 mmol.L^-1^, p < 0.001, for PLA, and 5.26 ± 0.78 mmol.L^-1^, p < 0.01 for SPD). The changes in glycemia were significantly more pronounced in PLA than in SPD (n = 13, p = 0.0002; Figure 2). Expressed as a percentage, the variations in glycemia were -16.2 ± 5.4 and -4.7 ± 2.9% for PLA and SPD, respectively (n = 13, p = 0.0007).

### Protocol 2: Standardized exercise

**Figure 3:**
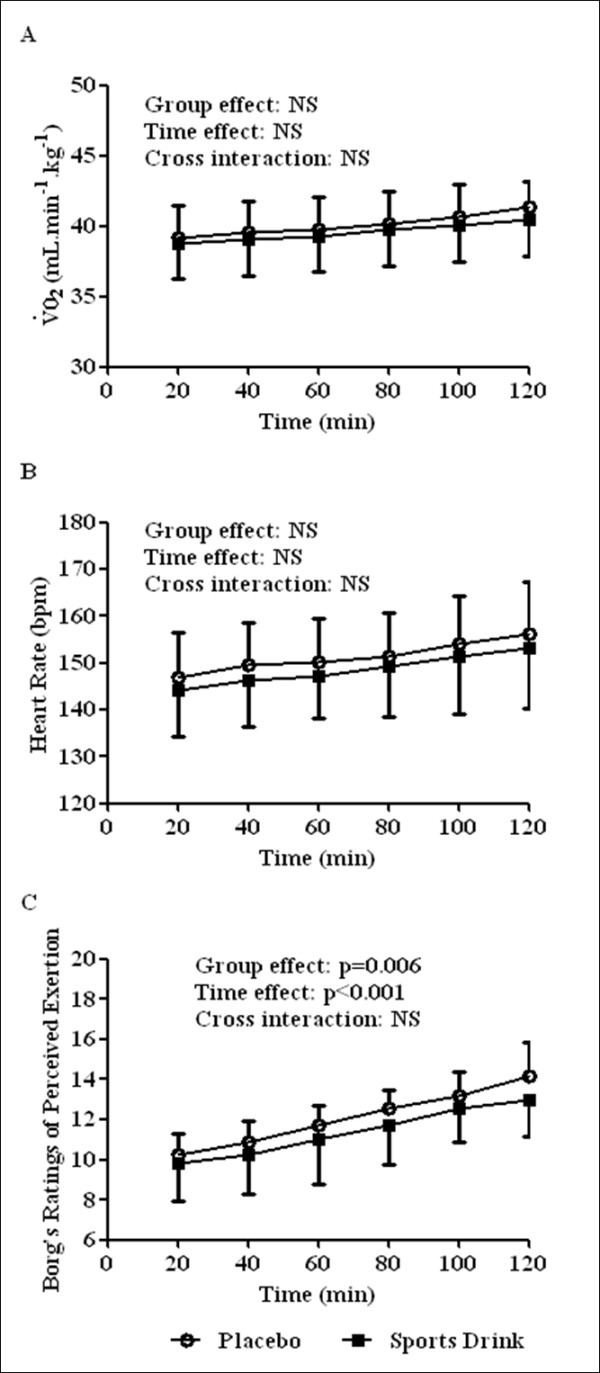
**Evolution of oxygen consumption (panel A), heart rate (panel B) and Borg's Rating of Perceived Exertion (panel C) during the standardized exercise protocol (protocol 2)**. Values are means ± SD.

**Figure 4:**
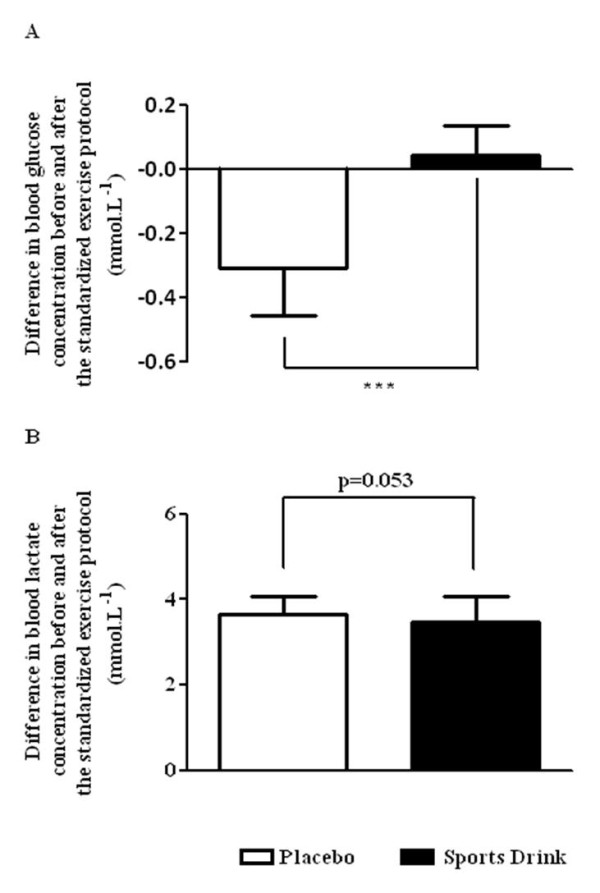
**Difference in blood glucose (panel A) and lactate (panel B) concentrations before and after the standardized exercise protocol (protocol 2)**. Values are means ± SD. *** p < 0.001.

For personal reasons, 2 subjects dropped-out of the study. The mean velocity during protocol 2 was 10.3 ± 0.6 km.h^-1 ^(n = 11). Changes in $\dot{\text{V}}{\text{O}}_{2}$, HR and RPE are shown in Figure 3. For $\dot{\text{V}}{\text{O}}_{2}$ and HR, no significant effect was observed (Figures 3A and 3B). A group and time effect was found for RPE (n = 11, group effect: p = 0.006, time effect: p < 0.001, cross interaction: NS; Figure 3C). For RER, no differences were found between the two conditions (data not shown). There was no difference in the glucose concentrations before exercise for PLA and SPD (5.40 ± 0.66 and 5.44 ± 0.67 mmol.L^-1^, respectively, n = 11). Glucose concentration decreased significantly after exercise in PLA (5.09 ± 0.60 mmol.L^-1^, n = 11, p = 0.001) but remained unchanged in SPD (5.48 ± 0.64 mmol.L^-1^, n = 11; Figure 4A). There was no difference in lactate concentration between the two conditions before exercise (1.65 ± 0.32 and 1.73 ± 0.42 mmol.L^-1 ^for PLA and SPD, respectively, n = 11). There was a tendency towards a lower blood lactate accumulation (post minus pre exercise values) in SPD (+3.48 ± 0.60 mmol.L^-1^) than in PLA (+3.65 ± 0.43 mmol.L^-1^) (n = 11, p = 0.053; Figure 4B) so that lactate concentration measured after exercise was significantly lower in SPD (5.20 ± 0.39 mmol.L^-1^) than in PLA (5.30 ± 0.35 mmol.L^-1^; n = 11, p = 0.01). The parameters of the neuromuscular functions are summarized in Table 2. The statistical analysis showed a deleterious effect of exercise on all the parameters of neuromuscular function and a higher decline in %VA and Db_100 _for the PLA condition compared with SPD. Although the alterations were lower in SPD than in PLA (-14% *vs*. -17%, respectively), the decreases in MVC were not significant between the two conditions.

**Table 2:** Neuromuscular variables before and after the standardized 120 min running exercise

	Pre		Post		(Post - Pre)/Pre values * 100 (%)		
	**PLA**	**SPD**	**PLA**	**SPD**	**PLA**	**SPD**	**p**

**MVC (Nm)**	116.9 ± 18.9	117.4 ± 20.1	96.7 ± 21.0	100.6 ± 19.6	-17 ± 11	-14 ± 10	0.55
**%AV**	0.97 ± 0.03	0.95 ± 0.04	0.88 ± 0.09	0.89 ± 0.09	-9 ± 7	-6 ± 6	0.04
**Db_100 _(Nm)**	52.4 ± 10.4	53.6 ± 10.2	45.0 ± 9.1	47.1 ± 7.3	-14 ± 9	-6 ± 5	0.04
**Pt (Nm)**	32.1 ± 7.4	32.9 ± 7.2	28.3 ± 7.1	28.5 ± 5.4	-12 ± 10	-13 ± 8	0.95
**CT (ms)**	100.35 ± 5.60	101.17 ± 3.83	94.22 ± 5.85	95.15 ± 6.01	-6 ± 3	-6 ± 4	0.94
**PPA (mV)**	17.74 ± 3.07	18.33 ± 2.70	15.08 ± 2.75	15.90 ± 2.49	-15 ± 6	-13 ± 2	0.80
**PPD (ms)**	8.74 ± 1.55	8.79 ± 1.28	7.94 ± 1.33	8.22 ± 1.20	-9 ± 6	-6 ± 5	0.52

MVC: Maximal voluntary contraction; %AV: maximal voluntary activation; Db_100_: Mechanical response to a double pulse at 100 Hz; Pt: Mechanical response to a single pulse; CT: contraction time (single twitch); PPA: M-wave peak-to-peak amplitude; PPD: M-wave peak-to peak duration.

Values are means ± SD. Statistical analysis was conducted on the (post - pre)/pre * 100 *i.e*., expressed in percentage (%) for PLA and SPD.

## Discussion

The main findings of the present study were that ingestion of the SPD containing CHOs (68.6 g.L^-1^), BCAAs (4 g.L^-1^) and caffeine (75 mg.L^-1^) immediately prior to and during a 2 h all-out or standardized exercise 1) increased running performance significantly, although to a moderate extent, 2) favored the maintenance of glycemia and 3) had variable effects on neuromuscular fatigue.

Performance, *i.e*. total distance over a 2 h running exercise, was significantly higher with SPD than in the placebo condition (22.31 ± 1.85 *vs*. 21.90 ± 1.69 km, respectively; p = 0.01). However, the increase in physical performance was rather small (+1.9%). Several reasons may explain this limited improvement. Firstly, because the subjects were not fasted (overnight), it can be hypothesized that initial muscle and liver glycogen stores were high, limiting the effects of SPD ingestion as has been previously shown [15]. Secondly, the importance of nutritional strategy during exercise of less than 2 hours seems to be limited [5,6,12]. The study by Coyle et al. [5] is of interest here. If the effect of CHO supplements improved performance by 33% (182 min PLA *vs*. 242 min in subjects using CHO supplements) during an exercise at 71% of V˙O2 max, it should be noted that glucose concentrations and CHO oxidation differed between the two conditions only after 80 min and 160 min of exercise, respectively. Moreover, in a recent meta-analysis of 72 studies, Karelis et al. [12] showed that the mean performance effect in studies with exercise durations higher than 2 h was significantly greater than in studies with exercise durations below 2 h. Our results agree with those of Jeukendrup et al. [6] who found that the positive effect of CHO supplements on performance was only 2.4% for a 1 hour exercise.

The results for neuromuscular function in the present study are variable. Firstly, both central fatigue and an index of peripheral fatigue (Db_100_) were significantly better preserved in the SPD than in the PLA condition. Along the same line, RPE was lower in SPD than in PLA (Figure 3C). However, although the alterations in MVC were lower in SPD than in PLA (-14% *vs*. -17%, respectively), the global index of neuromuscular fatigue (MVC) did not differ significantly between SPD and PLA. This lack of statistical difference is probably due to high inter-individual changes in MVC. An alternative explanation would be an alteration of excitation-contraction coupling or muscle fiber excitability. This may reduce the difference between SPD and PLA when MVC (*i.e*. trains of stimulations) is considered. However, excitation-contraction coupling and muscle fiber excitability do not seem to be affected by SPD as shown by the lack of difference in the M-wave characteristics and peak twitch changes between the two conditions.

In the present study, glycemia decreased during the all-out exercise (protocol 1) in both conditions, but the decrease was lower in SPD than in PLA. Furthermore, glycemia remained stable during the standardized event in SPD while it decreased in PLA (protocol 2). If SPD is helpful in maintaining glycemia, it should nevertheless be noted that the subjects were not hypoglycemic at the end of the exercise whatever the protocol or PLA condition. It has been postulated that the improved maintenance of blood glucose levels with the ingestion of glucose may not be a potential mechanism for improved performance during prolonged exercise [12]. However Nybo [35] showed that when blood glucose homeostasis was maintained by glucose supplementation, central fatigue seemed to be effectively counteracted and performance (average force production) increased. Of note is the fact that Nybo [35] detected central fatigue during a 2 min sustained maximal isometric contraction of the knee extensors but not during short contractions as in the present study. Glucose ingestion can stimulate the secretion of insulin and blunt the exercise-induced rise in both free fatty acids and free tryptophan and could consequently decrease central fatigue by attenuating the rise in brain 5-HT (serotonin) [36,37]. Of note, RPE was lower in SPD than in PLA (Figure 3C). Therefore, it is possible that in the present study, maintenance of blood glucose homeostasis indirectly acted via central fatigue to improve performance.

During sustained exercise, BCAAs are taken up by the muscles and their plasma concentration decreases. Decreased plasma BCAAs levels may lead to an increased plasma free tryptophan/BCAAs ratio, thus favoring the transport of tryptophan into the brain and consequently the synthesis of 5-HT. The subsequent production of serotonin could be responsible for the feeling of fatigue during and after sustained exercise. Nevertheless, it has been suggested that BCAAs supplementation during prolonged exercise may decrease central fatigue via reduced tryptophan uptake and 5-HT synthesis in the brain [4]. Indeed, because BCAAs and free tryptophan are transported into the brain by the same carrier system, BCCAs supplementation during exercise would decrease the plasma free tryptophan/BCAAs ratio. This would i) dampen the transport of tryptophan into the brain, ii) impede the subsequent synthesis and release of 5-HT, and consequently iii) reduce or delay the feeling of fatigue during and after sustained exercise

Caffeine ingestion might also affect central fatigue [38]. Human experiments have revealed that caffeine induces increases in central excitability, maximal voluntary activation, maximal voluntary force production and spinal excitability (for review, see Kalmar and Cafarelli [23]). The effect of caffeine on the central nervous system could be via its action on the blockage of adenosine receptors at concentrations in the micromolar range [23]. Stimulation of adenosine receptors induces an inhibitory effect on central excitability.

The present results show that concomitantly, CHOs, BCAAs and caffeine supplementation reduce central fatigue and RPE. Nevertheless, it is impossible in the present case to distinguish the individual contribution of each of them (CHOs, BCAAs and caffeine) in the positive effect of the sports drink on central fatigue and RPE.

The decrease in %VA (%VA changes were considered as indexes of central fatigue) is similar to the deficit observed in previous studies involving running exercises of comparable duration [39] and was only slightly, although significantly improved by the energy drink. The moderate influence on %VA could be explained by the fact that at least part of the decrease in %VA after prolonged running exercise has been attributed to the inhibitory effect if afferent fibers [40]. In particular, this could be due to reduced motoneurone excitability or to presynaptic inhibition, probably resulting from thin afferent fiber (group III-IV) signaling which may have been sensitized by the production of pro-inflammatory mediators produced during prolonged running exercise (*e.g*. [41]). Group III-IV afferent fibers may also contribute to the submaximal output from the motor cortex [42]. It is not known whether SPD had an effect on inflammation in the present study since no pro-inflammatory markers were assessed.

One limitation of this study is the fact that the volunteers were studied in a post absorptive state. This choice was made in an attempt to reproduce habitual race conditions since the main aim of this study was to investigate if ingestion of an association of CHOs, BCAAs and caffeine was useful in improving running performance. Other limitation concerns the lack of control of food intake before the trials. This may introduce variability between the trials and potentially between the conditions. Although the fact i) of performing the different conditions in a randomized order, ii) of starting every session at the same time of the day and iii) of instructing the subjects to replicate the same meal before each exercise session, allows to some extent limitation of variability between trials, it does not remove totally this variability. A careful attention should be paid in the future in the control of food intake before but also 2-3 days prior to testing.

## Conclusions

This study has shown for the first time that ingestion of a combination of CHOs (68.6 g.L^-1^), BCAAs (4 g.L^-1^) and caffeine (75 mg.L^-1^) immediately before and during a 2 h running exercise in standardized laboratory conditions significantly increased treadmill running performance by about 2% in trained subjects. Moreover, ingestion of a drink associating these components during a standardized 2 h running exercise maintained glycemia and significantly decreased RPE, central fatigue and an index of peripheral fatigue as compared to the placebo condition.

## Competing interests

Sébastien L Peltier is an employee of the company, Nutratletic, a subsidiary of Laboratoire Lescuyer. Jean-François Lescuyer is the general director for both companies. Other authors have no competing interests.

## Authors' contributions

SLP, GYM, PS, AG, MG, JFL and LM developed the study protocol. AG was the principle investigator and LM was the project leader of this study. AG, LF, LV and LM were in charge of the recruitment of the subjects. LV was in charge of data collection and management. JBM, MG, AG, GYM and LF participated in data collection. GYM was responsible for the central and peripheral fatigue measurements. Moreover, he also carried out the statistical analysis of theses specific variables. For other measures of fatigue, SLP was responsible for the statistical analysis. All authors have read and approved the final manuscript.
